# Nicotinic acetylcholine receptors in pain modulation

**DOI:** 10.3389/fphar.2026.1782495

**Published:** 2026-05-22

**Authors:** Junya Cheng, Junliang Chang, Shuai Li

**Affiliations:** Changchun Institute of Biological Products Co., Ltd., Changchun, China

**Keywords:** chronic pain, nicotinic acetylcholine receptors, nAChR subtypes, nAChR-targeted analgesics, translational pharmacology

## Abstract

Chronic pain is widely recognized as a major global health problem that affects approximately one-fifth of the adult population and is associated with significant physical, psychological, and socioeconomic burden. Current clinical analgesic strategies are mainly based on non-steroidal anti-inflammatory drugs, opioids, and adjuvant agents including antidepressants and antiepileptic drugs. However, these therapies are limited by insufficient efficacy in neuropathic pain, considerable adverse effects, and the risks of tolerance and addiction. Therefore, the development of safer and more effective non-opioid analgesics remains an important unmet clinical need. Nicotinic acetylcholine receptors (nAChRs) have been identified as promising targets for the development of next-generation analgesics because of their roles in pain signal transmission, neuroinflammation, and immune–neural interactions. nAChRs are pentameric ligand-gated ion channels composed of different subunit combinations, which give rise to multiple receptor subtypes with distinct expression patterns and functional properties. Increasing evidence suggests that specific nAChR subtypes, including α3β4, α4β2, α6β4, α9α10, and α7, participate in the regulation of inflammatory pain, neuropathic pain, and cancer-related pain. This review summarizes recent progress in the understanding of subtype-specific roles of nAChRs in pain regulation. The development of highly selective tool compounds, particularly α-conotoxin-derived peptides, is discussed together with current knowledge regarding the mechanisms associated with nAChR-mediated analgesia. Challenges related to clinical translation and potential therapeutic strategies are also considered.

## 1 Introduction

Pain is defined as an unpleasant sensory and emotional experience associated with actual or potential tissue damage. It represents a complex physiological and pathological process that includes both protective warning functions and pathological consequences (Raja et al., 2020). Epidemiological studies indicate that about 20% of adults worldwide experience persistent pain, and nearly 10% are newly diagnosed with chronic pain each year. This high prevalence reduces quality of life and places a substantial burden on healthcare systems and society, making pain a major global public health concern (Goldberg and McGee, 2011; Kuner and Flor, 2017). Although acute pain often reflects tissue injury and has physiological significance, persistent chronic pain may lead to several complications, including sleep disturbances, emotional disorders, and immune dysfunction. In some cases, chronic pain may develop into an independent disease entity, such as fibromyalgia. For this reason, the prevention and treatment of chronic pain have become important areas of medical research (Ayuso et al., 2022).

Clinical pain management commonly follows the World Health Organization three-step analgesic ladder, which recommends a stepwise approach from non-opioid analgesics to weak and then strong opioids (Auret and Schug, 2013). However, currently available analgesic therapies have several limitations. Non-steroidal anti-inflammatory drugs (NSAIDs) are mainly effective for inflammatory or nociceptive pain but show limited efficacy in neuropathic pain. Long-term use is limited by a ceiling effect and is associated with gastrointestinal injury, cardiovascular events, and hepatic or renal toxicity (Chang et al., 2021). Although opioid analgesics provide broad-spectrum pain relief, their use is associated with serious adverse effects, including addiction, tolerance, and respiratory depression, which have contributed to a global public health problem (Grosser et al., 2017). In addition, antidepressants and antiepileptic drugs used for neuropathic pain are often prescribed off-label. These agents show substantial interindividual variability in efficacy and are associated with significant side effects, which limits their clinical use (Hadley et al., 2016).

Given these limitations, there is a need to identify new analgesic targets and therapeutic strategies that provide effective pain control with improved safety. An ideal next-generation analgesic would have a clearly defined molecular target, high subtype selectivity, low risk of addiction and tolerance, and activity against pain conditions that are currently difficult to treat, particularly neuropathic pain. Nicotinic acetylcholine receptors (nAChRs) have received increasing attention as potential analgesic targets because they participate in pain signal transmission, neuroinflammatory regulation, and immune–neural interactions.

For this review, peer-reviewed studies on nAChRs in pain modulation and analgesic drug development were retrieved from PubMed and Web of Science. Publications from 2000 to 2026 were included, and searches applied combinations of keywords related to nAChRs (e.g., “nicotinic acetylcholine receptor,” “nAChR subtypes”), pain (e.g., “pain,” “neuropathic pain,” “inflammatory pain”), and pharmacology (e.g., “analgesia,” “agonist,” “antagonist,” “positive allosteric modulator”). Studies were selected based on their relevance to receptor subtype distribution, mechanisms of action, pharmacological properties, and evidence obtained from experimental or clinical pain models. Both preclinical and clinical studies were incorporated. Review articles were screened to provide context and to identify additional primary studies. Given the narrative scope of this review, formal systematic review methods were not applied. Instead, representative and frequently cited studies were prioritized to present a balanced overview of current knowledge and translational developments in this field.

## 2 Advances in the study of nicotinic acetylcholine receptors in pain modulation

Acetylcholine receptors (AChRs) are classified into two major types: nAChRs and muscarinic acetylcholine receptors (mAChRs). As central targets in basic pain research and analgesic drug discovery, AChRs have attracted increasing attention in recent years. Substantial progress has been made in elucidating their roles in pain modulation, providing important theoretical support and new directions for the development of novel analgesics.

nAChRs are ligand-gated ion channels that were first identified in 1973 in the electric organ of the ray *Torpedo marmorata* (Cartaud et al., 1973; Changeux, 2020). Structurally, nAChRs are pentameric receptors composed of five individual subunits arranged around a central pore. Upon activation, the ion channel opens and allows cation influx, leading to membrane depolarization (Davies et al., 2003). The nAChR family comprises multiple receptor subtypes, which differ in subunit composition, tissue distribution, and functional properties (Brockmöller et al., 2024). These differences enable distinct nAChR subtypes to play specific and critical roles in the initiation, transmission, and modulation of pain signals (Giastas et al., 2018).

nAChRs are characterized by rapid activation kinetics and can facilitate the release of inhibitory neurotransmitters, such as γ-aminobutyric acid (GABA), thereby suppressing pain signal transmission at the spinal level (Li et al., 2024a). Selective modulation of specific nAChR subtypes therefore offers a strategy for precise intervention in pain pathways and represents a promising alternative approach for clinical pain management. To date, 16 genes encoding nAChR subunits have been identified in the human genome, including α1–α7, α9, α10, β1–β4, δ, ε, and γ. Different combinations of these subunits give rise to a wide variety of functional nAChR subtypes (Gong et al., 2022; Hone and McIntosh, 2023; Millar and Gotti, 2009).

In the context of pain modulation, endogenous cholinergic signaling should be distinguished from pharmacological manipulation of nAChRs. Spinal acetylcholine is released mainly by a sparse population of choline acetyltransferase (ChAT)-positive dorsal horn interneurons, and supraspinal cholinergic pathways may also contribute to descending pain control (Naser and Kuner, 2018; Sullere et al., 2023). In contrast, many preclinical studies discussed below rely on exogenous agonists, antagonists, or positive allosteric modulators (PAMs), and thus primarily reflect pharmacological receptor engagement rather than physiological acetylcholine release. The following sections summarize subtype-specific roles of nAChRs in pain modulation and the representative evidence supporting them.

**FIGURE 1:**
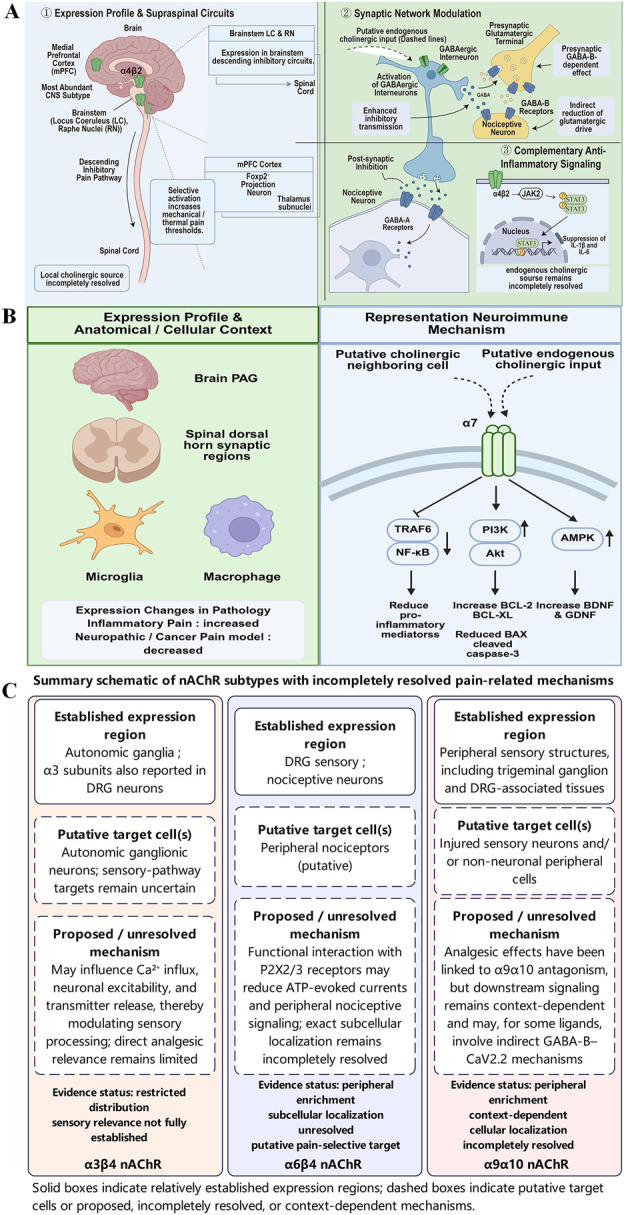
Subtype-specific schematic overview of nAChRs in pain modulation. **(A)** α4β2 nAChRs are shown as a mechanistically better-defined subtype, highlighting cholinergic input in descending inhibitory and cortical pain circuits, receptor expression in brainstem and mPFC-related networks, and downstream effects on inhibitory neurotransmission and inflammatory signaling that increase pain thresholds. **(B)** α7 nAChRs are shown as a neuroimmune and anti-apoptotic subtype, emphasizing expression in central and peripheral neuroimmune compartments and downstream anti-inflammatory, neuroprotective, and analgesic effects. **(C)** α3β4, α6β4, and α9α10 are summarized in a simplified panel showing known expression regions, putative target cells, and proposed or incompletely resolved pain-related mechanisms. Solid boxes indicate relatively established observations, whereas dashed boxes/arrows indicate putative, context-dependent, or incompletely resolved mechanisms.

nAChR subtypes differ markedly in their anatomical distribution, cellular targets, activation context, and downstream effects on nociceptive processing. To provide a clearer overview of these subtype-specific differences, the currently available evidence is summarized in Figure 1. The subtype-specific mechanisms discussed in the following sections are summarized in this figure set, with detailed mechanistic schematics for α4β2 and α7 and a simplified summary panel for α3β4, α6β4, and α9α10.

### 2.1 The α3β4 subtype

The α3β4 nAChR has a relatively restricted distribution and is best established in autonomic ganglia. Although α3 subunits, which may assemble with β4, have also been reported in dorsal root ganglion (DRG) neurons, the presence and functional significance of α3β4-containing receptors in peripheral sensory pathways remain to be fully established (Hone and McIntosh, 2023; Smith et al., 2013). Beyond sensory pathways, α3β4 nAChRs participate in ganglionic synaptic transmission, regulation of norepinephrine release, and neural circuits related to substance dependence, indicating a broader physiological role than pain modulation alone (Cippitelli et al., 2015; Li et al., 2020; Tang et al., 2011). Mechanistically, α3β4-related signaling may influence calcium influx, neuronal excitability, and transmitter release, thereby modulating sensory processing rather than directly encoding pain signals (Smith et al., 2013). Accordingly, selective antagonism or inhibition of α3β4 has been explored as a mechanistic strategy, although interpretation is complicated by the involvement of this subtype in autonomic and reward-related circuitry.

Several pharmacological tools have been used to probe α3β4 function. AuIB is a competitive antagonist with selectivity for α3β4-containing receptors; however, its best-characterized behavioral effects are reduction of nicotine-conditioned place preference and somatic withdrawal signs, whereas it does not alter nicotine-induced analgesia, suggesting a stronger role in reward-related processes than in direct antinociception (Elayouby et al., 2021; Jackson et al., 2013). Czon1107, a single-disulfide conopeptide from *Conus zonatus*, inhibits α3β4-containing receptors through a non-orthosteric allosteric mechanism and suppresses acetylcholine-evoked currents; it has also shown analgesic effects in preclinical models (Ma et al., 2022; Mohan et al., 2020). Overall, evidence supporting a direct analgesic role for α3β4 remains more limited than that for α4β2, α7, or α9α10 receptors, and no α3β4-targeted analgesic has progressed to clinical development (Elayouby et al., 2021; Jackson et al., 2013). Taken together, α3β4 appears mechanistically relevant to cholinergic regulation of sensory signaling, but remains a comparatively less validated and less translationally developed analgesic target than several other nAChR subtypes.

### 2.2 The α4β2 subtype

The α4β2 nAChR is the most abundant nicotinic receptor subtype in the mammalian central nervous system (Hone and McIntosh, 2023). It is widely distributed in monoaminergic nuclei involved in descending inhibitory pain pathways, including the raphe nuclei and locus coeruleus (Jensen et al., 2005). Beyond brainstem circuits, recent circuit-mapping studies show that α4β2 receptors are enriched in Foxp2^+^ projection neurons in the medial prefrontal cortex (mPFC), which relay signals to thalamic subnuclei involved in both sensory and affective aspects of pain (Xie et al., 2026).

Human PET imaging using [^18^F]XTRA (^18^F-JHU86428), a radiotracer developed for *in vivo* PET imaging of α4β2 nAChRs, has revealed increased α4β2 binding in cortical and limbic regions in patients with Parkinson’s disease (Mills et al., 2025). Although these studies were not performed in pain populations, the findings suggest that cortical α4β2 availability may change across chronic neurological conditions. This broad but circuit-specific expression pattern supports a role for α4β2 receptors in pain modulation, substance dependence, and mood regulation (Yu et al., 2019).

α4β2 nAChRs regulate nociceptive processing primarily by enhancing inhibitory signaling. In cortical and spinal circuits, α4β2 activation increases GABAergic transmission and can indirectly reduce glutamatergic drive; in brainstem pain-control circuits, α4β2-containing receptors are positioned to strengthen descending inhibitory tone (Hosur and Loring, 2011; Jensen et al., 2005). Studies in resected human temporal lobe epilepsy cortex showed that α4β2 activation increases GABA release from interneurons while indirectly reducing glutamate transmission through presynaptic GABA-B receptors (Martinello et al., 2025). This dual effect increases inhibitory signaling and reduces excitatory drive, which may contribute to spinal and cortical pain modulation.

Activation of this receptor can also suppress inflammatory mediators such as IL-1β and IL-6 through the JAK2–STAT3 pathway. Selective activation of α4β2 receptors in mPFC Foxp2^+^ neurons increases mechanical and thermal pain thresholds, and this effect disappears when these neurons are chemogenetically silenced (Xie et al., 2026). These findings suggest two complementary mechanisms involving neuronal network modulation and anti-inflammatory signaling.

Several pharmacological strategies have been used to target α4β2 receptors. These include partial agonists such as varenicline, full agonists such as ABT-594, and PAMs such as NS9283 (Bagdas et al., 2018; Ding et al., 2018; Hayashi et al., 2017). In human cortical tissue, the α4β2 PAM dFBr enhances acetylcholine-induced increases in inhibitory synaptic activity without directly activating the receptor, suggesting a strategy to enhance endogenous cholinergic tone while reducing receptor desensitization (Martinello et al., 2025). Pharmacological studies further indicate that ABT-594 exerts antinociceptive effects in several pain models, with evidence for predominantly supraspinal nicotinic mechanisms, and that its anti-allodynic activity can be potentiated by the α4β2 PAM NS9283 (Hayashi et al., 2017).

In experimental pain models, ABT-594 produced strong analgesic activity in inflammatory and neuropathic paradigms and advanced to phase II clinical evaluation. Varenicline has also shown adjunctive analgesic effects in a rat sciatic nerve repair model, where it reduced prostaglandin E_2_, IL-6, and substance P levels and improved histological indicators of nerve regeneration (Ozturk et al., 2026). Co-administration of the α4β2 PAM NS9283 enhances the analgesic efficacy of ABT-594 at tolerated doses without increasing emetic adverse effects (Ding et al., 2018).

Despite strong preclinical evidence, translation into clinical therapy has been limited. Development of ABT-594 was discontinued because of dose-dependent adverse effects, including vasoconstriction and elevated blood pressure (Hayashi et al., 2017). The broad central and autonomic distribution of α4β2 receptors limits dose flexibility and increases the risk of cardiovascular and gastrointestinal side effects. In addition, variability in receptor availability across pathological conditions may complicate dose–response relationships (Mills et al., 2025). Although PAM-based combination strategies may improve tolerability (Bagdas et al., 2018), long-term safety and the consequences of sustained cholinergic modulation remain insufficiently characterized. α4β2 receptors therefore remain among the most extensively studied but clinically difficult nicotinic targets for analgesic development.

### 2.3 The α6β4 subtype

The α6β4 nAChR is enriched in DRG sensory neurons and has emerged as an attractive non-opioid target for pain research. Current evidence suggests that α6β4-related expression is enriched in DRG nociceptive neurons, although its exact subcellular localization within these cells remains incompletely resolved (Zhang et al., 2026). This peripheral localization distinguishes α6β4 from more broadly distributed subtypes, such as α4β2, and has been proposed to support its potential as a pain-selective molecular target.

In mechanistic terms, α6β4-mediated analgesia differs from classical activation-driven nicotinic signaling pathways. Rather than enhancing inhibitory neurotransmission, α6β4 has been reported to interact functionally with P2X2/3 receptors on DRG nociceptors, resulting in cross-inhibition that reduces ATP-evoked currents and decreases nociceptive signaling in neuropathic models (Limapichat et al., 2014). This receptor–receptor coupling suggests a peripheral mechanism that may suppress excitatory purinergic signaling, indicating possible relevance in neuropathic pain states associated with ATP-dependent hypersensitivity.

Pharmacological investigation has been facilitated by the development of subtype-selective ligands. The α-conotoxin VnIB shows nanomolar potency (IC_50_ ≈ 5.3 nM) and high selectivity (>1,800-fold over α6β2), which allows selective inhibition of α6β4-mediated currents in DRG neurons (van Hout et al., 2019). The small-molecule agonist A-844606 (EC_50_ ≈ 0.16 μM) shows more than 100-fold selectivity relative to α4β2 and α3β4, supporting the pharmacological accessibility of this subtype (Leung, 2004). Structural information from cryo-electron microscopy studies has described the subunit arrangement and ligand-binding interfaces of human α6β4 receptors, facilitating structure-guided ligand development (Su et al., 2025). Species-specific differences in the β4 subunit influence ligand sensitivity and may affect translation from animal models to humans (Hone et al., 2021; 2018b).

Preclinical evidence has mainly been obtained from peripheral neuropathic pain models.

Functional studies in DRG neurons indicate that α6-containing nAChRs can counteract ATP-driven hyperexcitability through interaction with P2X2/3 receptors, supporting a potential role for this pathway in limiting peripheral nociceptive input in neuropathic conditions (Limapichat et al., 2014; Wieskopf et al., 2015). However, clinical translation remains limited, and no α6β4-selective agent has progressed to late-stage trials for pain indications.

Major limitations include the difficulty of achieving strict subtype selectivity due to the high sequence similarity with α3β4 and α6β2 (Zhang et al., 2026), as well as uncertainty related to peptide stability, drug delivery, and long-term modulation of sensory neuron function. Species-dependent differences in β4 residues further complicate extrapolation from rodent models to humans (Hone et al., 2021; 2018b). In summary, although α6β4 represents a peripherally localized and mechanistically characterized analgesic target, significant pharmacological and translational barriers remain.

### 2.4 The α9α10 subtype

The α9α10 nAChR has received increasing attention as a target in peripheral pain research. α9 and α10 subunits have been reported in peripheral sensory structures, including the trigeminal ganglion and, in some studies, DRG-associated tissues (Li et al., 2024a), whereas their dominant pain-relevant cellular localization remains incompletely resolved. This peripheral enrichment distinguishes α9α10 from more centrally expressed nicotinic subtypes and supports its consideration as a target for analgesic strategies with fewer central adverse effects.

In mechanistic terms, α9α10-related signaling has been proposed to influence peripheral nociceptive processing, potentially including effects on neuropeptide signaling such as substance P and CGRP, although the direction and cellular context of this regulation remain incompletely defined. Antagonism of α9α10 nAChRs has been reported to reduce neuropathic pain-related behaviors in several rodent models (Satkunanathan et al., 2005). Electrophysiological studies have suggested context-dependent effects of α9α10-related signaling in injured sensory neurons, potentially involving modulation of calcium channel activity, indicating functional heterogeneity across experimental settings (Hone et al., 2018a). For some analgesic conopeptides initially studied as α9α10 ligands, an alternative mechanism has been proposed in which the peptide engages GABA-B-dependent signaling and thereby inhibits CaV2.2 channels in sensory neurons. This mechanism appears to be peptide-dependent rather than a universal consequence of α9α10 antagonism (Li et al., 2024b). The observation that both antagonists and agonists can produce anti-inflammatory or analgesic effects reflects the complex nature of α9α10 signaling and may involve indirect pathways such as GABA-B-mediated inhibition of CaV2.2 channels.

A range of pharmacological tools has enabled functional characterization of this receptor subtype. The α-conotoxin Vc1.1 was among the first α9α10-selective antagonists examined and showed analgesic activity in the chronic constriction injury (CCI) model (Satkunanathan et al., 2005). However, reduced affinity for the human receptor compared with rodent orthologs limited its clinical efficacy (Yu et al., 2013). Species differences have been linked to structural variation within the ligand-binding interface; residue 59 in the α9 subunit determines differential binding of RgIA between rat (Thr59) and human (Ile59) receptors (Azam and McIntosh, 2012). Subsequent peptide optimization, including [S4Dap]Mr1.1, GeXIVA, RgIA analogues, and RgIA4, improved stability and maintained antinociceptive activity in rodent neuropathic models (Di Cesare Mannelli et al., 2014; Li et al., 2016; Liang et al., 2022; Luo et al., 2015). RgIA4 has also been reported to promote structural nerve recovery and to require CD3^+^ T cells for full analgesic activity (Azam et al., 2024; Huynh et al., 2022; Romero et al., 2017). N-terminally modified GeX-2 further improved serum stability and, in this peptide series, supported a GABA-B–CaV2.2–dependent mechanism of neuropathic pain relief (Li et al., 2024b). Small-molecule antagonists such as ZZ-204G and ZZ1-61c have also shown efficacy in mechanical hyperalgesia and paw withdrawal models (Holtman et al., 2011; Wala et al., 2012).

Preclinical studies across peripheral neuropathic pain models support antinociceptive effects of α9α10 blockade, including in CCI, mechanical hyperalgesia, and inflammatory paradigms (Holtman et al., 2011; Satkunanathan et al., 2005). Some agonist-like ligands acting at α9-containing and/or related non-neuronal nicotinic receptor assemblies, including pCF3-diEPP, have also shown anti-inflammatory activity through suppression of lipopolysaccharide-induced cytokine release, including IL-6 in primary mouse macrophages and IL-6, TNF-α, and IL-1β in whole human blood cultures (Andleeb et al., 2025; Richter et al., 2022), although the relative contribution of α9α10 compared with other nicotinic subtypes, such as α7, remains debated (Garcia et al., 2022; Rowley et al., 2010; Sgard et al., 2002).

With respect to translation, early clinical evaluation of Vc1.1 did not reproduce the analgesic effects observed in preclinical studies, largely due to insufficient engagement of the human receptor (Yu et al., 2013). Later peptide optimization improved pharmacokinetic stability and receptor selectivity in preclinical systems, but clinical evidence in human pain populations remains limited. The involvement of T cell–dependent mechanisms further complicates identification of appropriate patient populations and biomarkers.

Major limitations include cross-species pharmacological differences related to structural variation in the α9 ligand-binding interface (Azam and McIntosh, 2012), variability in immune-dependent mechanisms, and challenges associated with peptide stability, delivery, and long-term administration. Context-dependent effects of agonists and antagonists also complicate mechanistic interpretation. Although α9α10 antagonists consistently show analgesic activity in rodent neuropathic pain models, the predictive value of these models for human chronic pain remains uncertain. Representative studies used different administration routes, including intramuscular administration for GeXIVA-based peptides and subcutaneous administration for RgIA4 in chemotherapy-induced cold allodynia models. Most studies rely on acute nerve injury paradigms such as the CCI model, which capture peripheral sensitization but only partly reflect the neuroimmune and affective components of chronic pain. The limited clinical performance of Vc1.1 illustrates this translational gap and indicates the need for experimental models that better incorporate immune modulation, chronic disease progression, and species-specific receptor pharmacology.

### 2.5 The α7 subtype

The α7 nAChR has been widely investigated among nicotinic receptor subtypes (Papke and Lindstrom, 2020). It is expressed throughout the central and peripheral nervous systems and is also present in immune cells, including microglia and macrophages. High levels have been reported in regions associated with descending pain modulation, such as the periaqueductal gray (Umana et al., 2017). Imaging and *in situ* hybridization studies have confirmed its presence in microglia and synaptic regions of the spinal dorsal horn, which suggests involvement in neuroimmune regulation (Hone and McIntosh, 2023; Tan et al., 2025; Wong et al., 2018; Zhou et al., 2022). Under different pathological conditions, α7 expression has been reported to change, with increased levels observed in inflammatory pain and reduced expression reported in neuropathic and cancer pain models (Zhang et al., 2021; Zhou et al., 2022). Outside neuronal compartments, α7 nAChRs also form part of the non-neuronal cholinergic system and the cholinergic anti-inflammatory pathway. Interaction with the chaperone protein RIC-3 has been linked to regulation of macrophage polarization and age-related inflammatory processes (Chiappelli et al., 2025).

Unlike ionotropic subtypes that directly regulate nociceptive transmission, α7 nAChRs appear to produce analgesic effects mainly through regulation of inflammatory and neuroimmune signaling (Bouzat et al., 2018). Activation of α7 has been reported to inhibit the TRAF6/NF-κB pathway, activate AMPK signaling, and affect mitochondrial function (Jiang et al., 2021; Zhang et al., 2021). In microglia, nicotine and the α7-preferring partial agonist GTS-21 inhibit LPS-induced release of IL-6, IL-17, MCP-1, and RANTES through an α7/PI3K-dependent mechanism, and these effects can be reversed by the antagonist MLA. At the same time, increased expression of BDNF and GDNF has been reported (Qin et al., 2023). α7 activation has also been associated with reduced astrocytic and microglial activation and decreased production of IL-1β and TNF-α (Guerriero et al., 2025; Teng et al., 2025). In addition, α7 signaling has been linked to activation of anti-apoptotic pathways through PI3K/Akt-dependent increases in Bcl-2 and Bcl-xL and reduced expression of Bax and cleaved caspase-3, which may contribute to neuroprotection and limit the development of persistent pain states (Mugayar et al., 2023). Metabolic changes in macrophages and reduced HMGB1 release have also been reported, further indicating that α7 may participate in regulation of inflammatory pain conditions (Chen et al., 2025; Yang et al., 2025).

From a pharmacological perspective, strategies targeting α7 include orthosteric ligands such as the α7-preferring partial agonist GTS-21 and the agonist PNU-282987, PAMs including PNU-120596 and PAM-4, and silent agonists. Silent agonists are ligands that bind α7 nAChRs and stabilize non-conducting receptor states without producing classical ion channel activation, such as R-47 (Bagdas et al., 2021; Ji et al., 2025; Papke et al., 2023; Toma et al., 2019). In rodent models of inflammatory, neuropathic, and cancer pain, these compounds have been reported to reduce mechanical allodynia, thermal hyperalgesia, and markers of neuroinflammation. A systematic review also indicates that α7 agonists and PAMs produce analgesic effects in several peripheral neuropathic pain models, although differences between pain modalities have been observed for some compounds (Montigné and Balayssac, 2023). Genetic studies provide additional support for these findings, as α7 knockout mice show increased nociceptive behaviors, whereas gain-of-function hypersensitive α7 knock-in models, including the L250 T mutation model, exhibit reduced nociceptive responses (Zhang et al., 2021).

Despite strong preclinical evidence, translation to clinical therapy remains limited, and no α7-targeted analgesic has yet received regulatory approval. Receptor desensitization and indirect anti-inflammatory mechanisms may limit sustained clinical efficacy. Failures during late-stage development suggest that anti-inflammatory effects observed in experimental models do not always predict clinical efficacy. This inconsistency may partly reflect species-dependent pharmacology and the indirect nature of α7-related analgesic mechanisms. The widespread distribution of α7 receptors in the central nervous system and their Ca^2+^ permeability also raise concerns regarding off-target effects, receptor desensitization, and safety during prolonged activation. In addition, disease-specific changes in receptor expression, variability in immune responses, and potential sex-dependent differences complicate dose selection and patient stratification (Tao et al., 2025; Zhou et al., 2022). For these reasons, PAMs are increasingly considered a potentially more feasible strategy, as they enhance endogenous cholinergic signaling while avoiding continuous receptor stimulation.

### 2.6 Core mechanisms of nAChR-Mediated analgesia

To facilitate cross-subtype comparison, the major features of nAChR subtypes involved in pain modulation are summarized in Table 1.

**TABLE 1:** Major nAChR subtypes implicated in pain modulation and their pharmacological characteristics.

Subtype	Main distribution	Analgesic mechanism	Representative ligands	Mode of action	Experimental evidence	Translational status	Major limitations
α3β4	Autonomic ganglia; DRG-associated sensory pathways (proposed)	Modulation of neuronal excitability and neurotransmitter release	AuIB, Czon1107	AuIB—competitive antagonist; czon1107—non-orthosteric allosteric inhibitor	Limited pain models	AuIB—preclinical tool only Czon1107—preclinical lead only	Role overlaps with reward and autonomic circuits
α4β2	CNS, descending pain pathways	Enhances GABAergic inhibition, reduces glutamatergic transmission	ABT-594, varenicline, NS9283	ABT-594—agonist; varenicline—partial agonist; NS9283—PAM	Extensive rodent evidence	ABT-594—phase II, discontinued; varenicline—approved for smoking cessation, no pain program; NS9283—preclinical only	Narrow therapeutic window, cardiovascular side effects
α6β4	DRG nociceptors	Cross-inhibition with P2X2/3 receptors	VnIB	VnIB—selective antagonist	Neuropathic pain models	VnIB—preclinical tool/scaffold only	Subtype selectivity and species differences
α9α10	Peripheral sensory structures (including trigeminal ganglion; DRG-associated tissues proposed)	Proposed modulation of neuropeptide signaling; peptide-dependent alternative GABAB–CaV2.2 mechanism	Vc1.1, RgIA4, GeXIVA	Vc1.1—antagonist; RgIA4—antagonist; GeXIVA—antagonist	Strong preclinical evidence in rodent neuropathic pain models	Vc1.1/ACV1—publicly reported phase I/IIa history, not advanced; RgIA4—preclinical only; GeXIVA—preclinical lead only	Species-dependent pharmacology
α7	CNS and immune cells	Cholinergic anti-inflammatory pathway	GTS-21, PNU-282987, PAM-4	GTS-21—α7-preferring partial agonist; PNU-282987—full agonist; PAM-4—pam	Broad preclinical models	GTS-21—phase II outside pain indications; PNU-282987—preclinical only; PAM-4—preclinical only	Indirect mechanism and desensitization

Although nAChR subtypes show substantial differences in expression patterns and functional characteristics, their analgesic effects are largely related to their activity as ligand-gated ion channels (Zouridakis et al., 2009). After activation, nAChRs act as non-selective cation channels that allow Na^+^ and Ca^2+^ entry together with K^+^ efflux. This process can alter membrane potential and intracellular signaling pathways and influence neuronal excitability and inflammatory activity (Bader et al., 2017; Borroni and Barrantes, 2021). Ion influx can directly modify the firing patterns of nociceptive neurons and may reduce abnormal excitability. In addition, Ca^2+^ functions as a second messenger that regulates release of neurotransmitters such as glutamate, GABA, and dopamine, which can modify pain transmission pathways indirectly. Some receptor subtypes, including α7 and α9α10, also reduce neuroinflammatory responses through regulation of immune cells, which has been described as a combined neural and immune mechanism of analgesia (Chen et al., 2023).

At the peripheral level, several nAChR subtypes influence the activity of primary nociceptors. For example, α6β4 receptors identified in dorsal root ganglion neurons may reduce ATP-dependent nociceptive signaling through functional interactions with P2X2/3 receptors, thereby limiting peripheral nociceptive transmission. In addition, α9α10-related signaling has been proposed to influence peripheral nociceptive processing, potentially including effects on neuropeptide signaling, although the direction and cellular context of this regulation remain incompletely defined. Antagonism of α9α10 nAChRs has been reported to reduce pain-related behaviors in several rodent neuropathic pain models, although the precise contribution of neuropeptide signaling to these effects remains incompletely resolved.

Within the spinal dorsal horn, nAChRs influence the balance between excitatory and inhibitory synaptic activity. Activation of α4β2 receptors increases inhibitory GABAergic signaling and can reduce presynaptic glutamate release, which decreases ascending nociceptive input. At the same time, α7 receptors expressed on microglia participate in the cholinergic anti-inflammatory pathway. Activation of this pathway reduces the production of pro-inflammatory cytokines and may contribute to regulation of neuroinflammation associated with chronic pain.

At the supraspinal level, nAChRs contribute to descending inhibitory pathways that arise from several brainstem nuclei. Activation of receptors in regions including the locus coeruleus and raphe nuclei promotes release of monoaminergic transmitters such as norepinephrine and serotonin. These transmitters act within the spinal cord to suppress nociceptive transmission and strengthen endogenous analgesic control.

## 3 Discussion

The evidence summarized in this review indicates that several nAChR subtypes, including α4β2, α7, α9α10, and α6β4, contribute to pain regulation through distinct mechanisms. However, despite increasing experimental support for nAChRs as potential analgesic targets, several conceptual and methodological issues remain unresolved. First, many mechanistic studies examine individual receptor subtypes separately, whereas pain processing *in vivo* likely involves coordinated signaling across multiple cholinergic and non-cholinergic pathways. Second, most preclinical studies rely mainly on rodent neuropathic pain models, which may not fully represent the multidimensional characteristics of human chronic pain, including emotional and cognitive components. These limitations indicate the need for integrative approaches that integrate circuit-level analysis, human tissue studies, and translational biomarkers.

Research on acetylcholine receptors in pain has progressively shifted from descriptive observations toward subtype-specific mechanisms, selective pharmacology, and translational relevance. The following discussion highlights recent progress in tool-compound development, mechanistic studies, and clinical translation.

### 3.1 Development of subtype-selective tool compounds

Subtype-selective tool compounds are essential for investigating the functional roles of individual nAChR subtypes in pain regulation and for supporting the development of targeted analgesics (Do et al., 2025). Because many nAChR subtypes share high sequence similarity, particularly within ligand-binding domains, traditional small-molecule ligands often show limited subtype selectivity. As a result, off-target activity may occur, which can complicate mechanistic interpretation and increase safety concerns in translational studies. The development of highly selective subtype-specific ligands has therefore become an important focus in this field.

Among current approaches, conotoxins and their engineered derivatives provide a valuable source of subtype-selective ligands (George et al., 2024). Conotoxins are peptides derived from cone snail venom and naturally display strong receptor subtype selectivity (Aridoss et al., 2021). Through structural modification and rational design of α-conotoxins, several high-affinity ligands have been developed for specific nAChR subtypes (Bekbossynova et al., 2021). For example, the Hα6/α3Hβ4 mutant developed for the α6β4 subtype shows more than a tenfold increase in binding affinity compared with the native peptide while displaying minimal activity toward other subtypes such as α3β4 and α9α10. This ligand has provided a useful experimental tool for studying the role of α6β4 nAChRs in neuropathic pain (Dash and Li, 2014; Hone et al., 2018b; van Hout et al., 2019).

In addition to natural peptide derivatives, synthetic peptides designed to target specific receptor subtypes have also shown potential. For instance, the synthetic peptides PMP-072 and PMP-311 selectively activate α7 nAChRs and enhance the cholinergic anti-inflammatory pathway (van Maanen et al., 2015; Shelukhina et al., 2023). Their *in vivo* half-life is longer than that of many native peptides, and stable analgesic effects have been reported in inflammatory pain models. These compounds provide useful lead structures for further development of α7-targeted therapies.

However, despite their high subtype selectivity, peptide-based ligands face several limitations that complicate clinical translation. Restricted routes of administration, limited biological stability, and relatively high production costs remain important concerns. Improving pharmacokinetic properties while maintaining receptor selectivity therefore remains a key task in the transition from experimental tool compounds to therapeutic agents.

### 3.2 Clinical potential and translational barriers of nAChR-Targeted analgesics

The development of acetylcholine receptor (AChR)-targeted analgesics has gained increasing attention, as current pain therapies are limited by opioid dependence and the long-term toxicity of nonsteroidal anti-inflammatory drugs (Knezevic et al., 2017; Wu et al., 2024). This approach is supported by the subtype-specific roles of AChRs in pain signaling and neuroinflammatory regulation, as well as by advances in peptide engineering, small-molecule design, and drug delivery systems (Giraudo et al., 2023; Whitehead et al., 2021; Zhao et al., 2019). From a clinical perspective, these agents may offer additional treatment options for conditions with limited therapeutic choices, including neuropathic and cancer-related pain, and may reduce systemic adverse effects and addiction risk (Bele et al., 2024; Hone and McIntosh, 2018; Luo and Huang, 2024; Xu et al., 2022).

In recent years, several candidate drugs targeting specific nAChR subtypes have advanced to late-stage preclinical studies or early clinical trials (Brinzeu et al., 2024; Shao et al., 2021). Initial evidence of analgesic activity and acceptable short-term safety has been reported, particularly for peptide-based ligands such as α-conotoxin derivatives, which exhibit high subtype selectivity and relatively low abuse liability (Huang et al., 2019). However, most available data are limited to early-phase studies, and long-term efficacy and safety in human populations have not yet been established.

Several factors continue to limit clinical translation. Previous failures of α4β2-and α7-targeted compounds suggest that strong efficacy in preclinical models does not necessarily predict clinical success (Bertrand et al., 2010; Lassota et al., 2025; Toma et al., 2020). Species-dependent differences in receptor pharmacology remain a major concern, as ligands optimized in rodent systems may exhibit reduced potency or altered efficacy at human receptors (Margiotta et al., 2022). In addition, blood–brain barrier penetration represents a trade-off between efficacy and safety, as central exposure may increase the risk of neuropsychiatric or autonomic adverse effects.

Receptor-specific factors further complicate clinical translation. For example, α7 signaling exhibits context-dependent effects, with protective roles reported in some conditions and detrimental effects observed in infectious or inflammatory settings (Chi et al., 2011; Dash et al., 2016; He et al., 2021). Off-target activation of related receptor subtypes, particularly those widely expressed in central and autonomic systems, may reduce the therapeutic window (Bertrand et al., 2010). In addition, sustained receptor activation may result in desensitization or functional tolerance, and variability in immune status across patients may affect treatment responses.

Future development may focus on strategies to improve selectivity and safety. Targeting peripherally enriched subtypes such as α6β4 and α9α10 may reduce central adverse effects. The use of PAMs or biased ligands may enhance endogenous signaling while limiting receptor desensitization. Combination therapies that combine nAChR-targeted agents with existing analgesics may also improve overall efficacy.

Although AChRs are not universal targets for pain control, they provide a mechanism-based approach for the selective modulation of pain pathways. Continued advances in receptor pharmacology, ligand design, and translational models are required to support the development of effective and clinically applicable nAChR-targeted analgesics.

## 4 Conclusion

nAChRs are involved in pain regulation at peripheral, spinal, and supraspinal levels. Different receptor subtypes have been reported to contribute to analgesic effects through several mechanisms, including the modulation of nociceptor excitability, the regulation of spinal inhibitory circuits, and the activation of descending monoaminergic pathways. Current evidence also suggests that nAChRs participate in neuroimmune interactions related to chronic pain.

Several strategies have been investigated for the development of nAChR-targeted analgesics, including subtype-selective ligands, peptide derivatives such as conotoxins, small-molecule modulators, and improved drug delivery approaches. Among these subtypes, α4β2 and α7 receptors have been extensively studied in central pain pathways, whereas α9α10 and α6β4 receptors show more restricted peripheral expression and may represent potential targets for more selective analgesic strategies. Peripherally enriched subtypes, such as α9α10 and α6β4, may provide opportunities for more selective pain intervention, whereas centrally expressed receptors including α4β2 and α7 are associated with broader neuromodulatory functions.

However, translation of these findings into clinical practice remains limited. Species differences, receptor desensitization, limited subtype selectivity, and challenges in drug delivery continue to limit therapeutic development. Further progress in receptor pharmacology, ligand design, and translational research models is required to support the development of safer and more effective nAChR-targeted analgesics.
